# Facteurs associés à la non observance thérapeutique des sujets adultes infectés par le VIH sous antirétroviraux dans un hôpital de référence à Douala

**DOI:** 10.11604/pamj.2015.20.412.5678

**Published:** 2015-04-27

**Authors:** Emmanuel Noel Essomba, Dieudonné Adiogo, Danielle Christiane Kedy Koum, Baudouin Amang, Leopold Gustave Lehman, Yves Coppieters

**Affiliations:** 1 Faculté de Médecine et des Sciences Pharmaceutiques de Douala, Cameroun; 2 Comité National de Lutte contre le SIDA- Cameroun; 3 Ecole de santé publique, Université Libre de Bruxelles, Belgique (ULB)

**Keywords:** Antirétroviraux, nonobservance, VIH/SIDA, Cameroun, antiretrovirals, nonadherence, HIV/AIDS, Cameroon

## Abstract

**Introduction:**

Le succès du traitement antirétroviral repose sur l'observance. Elle est nécessaire pour réduire la mortalité, diminuer le risque de résistance et restaurer l'immunité. Cette étude a pour but d'identifier et analyser les différents facteurs associés à la non observance thérapeutique des patients infectés par le VIH sous traitement antirétroviraux à l'hôpital de référence Laquintinie de Douala.

**Méthodes:**

Il s'agit d'une étude transversale et analytique effectuée de mars à juin 2014. La non observance est mesurée à travers les déclarations du patient et sur consultation des registres de renouvellement des ordonnances. Etaient non observant, ceux ayant consommé moins de 95% de médicaments et ceux ne s’étant pas présentés pour le renouvellement de l'ordonnance. L'analyse bivariée et le modèle de régression logistique ont été utilisés pour la détermination des facteurs associés à la non observance.

**Résultats:**

Au total, 524 patients ont été enrôlés dans l’étude;l’âge moyen était de 43,0 ± 10,7 ans et le sexe ratio H/F de 0,54. De ces patients, 49,0% étaient non observant, majoritairement des femmes (61,9%). Les principales raisons avancées de la non observance sont: l'oubli (32,9%), la rupture de médicaments (14,0%), les occupations (12,8%). Les personnes veuves(IC 95% OR= 1,31-5,22, p= 0,006), la consommation des excitants (IC 95%, OR= 2,30-6,90, p= 0,0001) et la présence d'infection opportuniste (IC 95%, OR= 1,41-17,54, p= 0,01) ont fortement été associés à la non observance.

**Conclusion:**

Le taux d'observance était faible, lié à plusieurs facteurs. Des mesures sont nécessaires pour résoudre ce problème, y compris des stratégies tendant à l'amélioration du soutien psycho-social, et la limitation des ruptures de stock de médicaments. La recherche qualitative est souhaitée pour comprendre les raisons de la non observance afin de mettre au point des interventions fondées sur des données probantes.

## Introduction

Le syndrome de l'immunodéficience acquise (SIDA) s'accompagne d'un déficit immunitaire et des manifestations cliniques variables, notamment, des infections opportunistes, des néoplasies lymphoprolifératives et des troubles neurologiques [1]. En 2012, 34 millions de personnes vivaient dans le monde avec le virus de l'immunodéficience humaine (VIH) [2]. L'Afrique subsaharienne reste l'une des régions les plus gravement touchées avec près d'un adulte sur 20 (4,9%), ce qui représente 69,0% des personnes vivant avec le VIH dans le monde [2]. De nombreux pays ont mis sur pied des stratégies de lutte afin de stopper l'avancée de l'infection et améliorer le confort de vie des malades. Celles-ci visent la disponibilité de molécules antirétrovirales efficaces, dont l'association a permis de réduire significativement la mortalité par le VIH [3, 4]. En effet, ces substances entraînent une chute de la charge virale avec pour conséquence une restauration de l'immunité [5]. Les multithérapies antirétrovirales ont modifié la prise en charge de l'infection à VIH et transformé la perception du SIDA qui est devenu une maladie chronique avec laquelle on peut vivre [6, 7]. De ce fait, de nouvelles problématiques liées à la chronicité de la maladie sont apparues, et notamment celle de l'observance thérapeutique des patients. L´observance permet de décrire « le degré d´adéquation entre le comportement du patient, en termes de prise de médicaments, de prescriptions et les recommandations médicales» [6]. L´emploi de ce terme inclut la notion d´adhésion qui désigne le degré d´acceptation du patient vis-à-vis de la prescription. Aussi, pour certains auteurs, une observance au traitement de 95% s'avère indispensable pour le succès thérapeutique [8, 9], ceci dans le but d'atteindre les objectifs thérapeutiques qui sont: prolonger la vie, diminuer la fréquence des affections opportunistes, arrêter ou ralentir rapidement et durablement la réplication virale, restaurer ou améliorer l´immunité de la personne infectée [10, 11]. Le danger d'une mauvaise observance est l’émergence des résistances aux virus [12]. L´observance est appréciée entre autres par le comptage des comprimés restant, dans les systèmes ou le patient est appelé á s´approvisionner en médicaments auprès d´une même pharmacie [12]. Le taux de renouvellement des ordonnances est apparu être un bon indicateur d´observance, sensible à la durée ou la complexité du traitement antirétroviral (TAR) [13]. Plusieurs études se sont appuyées sur ces différentes méthodes. Orrell et al. ont trouvé une non observance (NO) par la méthode de renouvellement des ordonnances de 93,5%, dans une cohorte de 289 personnes infectés par le VIH (PVVIH) en Afrique du Sud et vivant dans une extrême pauvreté [14]. A Dakar, une étude a mesuré l´observance par la méthode de comptage des restes de médicament à chaque visite, sur une période de 12 mois et sur un échantillon de 158 adultes infectés par le VIH. Elle relate une NO de 91% [15]. Les données sur l'observance thérapeutique sont peu nombreuses au Cameroun. A Dschang en 2010, Mbopi-Kéou et al. ont trouvé une NO de 48,7% [16]. Or il est totalement admis que la réussite aux traitements antirétroviraux (TAR) dépend du degré d'observance à ces traitements. L'amélioration des connaissances vis-à-vis de cette problématique est un atout dans la prise en charge des PVVIH. La présente étude s'est fixée pour objectifs: (i) de déterminer la prévalence de la NO des personnes vivant avec le VIH (PVVIH) sous antirétroviraux à l'hôpital Laquintinie de Douala (HLD), (ii) de déterminer les caractéristiques socio-démographiques des patients NO et (iii) d'analyser les facteurs entrainant la NO dans un hôpital de référence national.

## Méthodes

Nous avons mené une étude transversale et analytique, qui s'est déroulée de mars à mai 2014 au centre de traitement agrée (CTA) de l'hôpital du jour (HDJ), de l'HLD, en charge du suivi de personnes vivant avec le VIH/SIDA. La ville de Douala est la capitale économique du Cameroun, ville cosmopolite dans laquelle cohabitent plus de trois millions d'habitants avec des structures hospitalières permanemment sollicitées pour des pathologies diverses. L'hôpital Laquintinie de Douala est une structure de référence nationale, disposant en son sein d´une structure spécialisée dans la prise en charge des PVVIH. Il s'agit d'un CTA selon la classification des niveaux du système de santé au Cameroun, ayant une file active d'environ 5000 patientsau moment de l’étude [17].

**Population de l’étude:** ce travail concerne tous les patients de plus de 15 ans infectés par le VIH, sous TAR depuis au moins un mois et pris en charge à HDJ de l'HLD et auprès desquels un accord a été obtenu à travers un consentement éclairé. L’échantillon de cette étude est constitué de sujets non hospitalisés venus pour le renouvellement de leurs ordonnances ou pour leurs visites médicales de routine. Pour les patients de 15 à 18 ans, une autorisation parentale était requise. Tous les patients remplissant les critères d'inclusion ont été systématiquement enrôlés. Le calcul de la taille de l’échantillon a fait appel à la formule de Lorentz [18]. Selon une étude menée à Dschang au Cameroun en 2010, la prévalence de la non observance était de 48,7% [16]. Pour des raisons d'approximation, la même prévalence a été utilisée pour le calcul de cette taille. Aussi, la taille minimale requise était de 384 patients.

**Collecte des données:** la collecte des données s´est faite á l`aide d`un questionnaire pré testé. Les données ont été collectées suite à des entretiens confidentiels de 10 à 15 minutes dans des lieux isolés du public. A l'aide d'un code d'identification inscrit sur le carnet du malade, son dossier était retrouvé. Le malade ainsi identifié était interviewé sur la base de ce questionnaire. L'observance thérapeutique a été évaluée de deux manières: i)- soit les patients avaient manqué plus d'une prise de médicament au cours des 7 derniers jours précédant le début de l'enquête pour les protocoles à trois prises journalières; ou les patients avaient manqué pendant une semaine ou plus leur traitement durant le mois précédant le début de l'enquête ou depuis l´initiation thérapeutique [19]; ii)- soit par le rapport entre le nombre d'ordonnances dispensées et la quantité théorique d'ordonnances attendues (correspond au nombre de mois de suivi du traitement). Le patient était déclaré NO lorsque ce dit rapport était inférieur à 0,95 [8, 9]. Les variables indépendantes suivantes ont été récoltées sur base du questionnaire puis des dossiers médicaux: les données démographiques (l’âge, le sexe, la religion, l'ethnie, le statut matrimonial, le lieu d'habitation, le niveau d’étude), les données socio-économiques (la profession, les activités génératrices de revenus), les avis sur les rapports avec le personnel médical et le fonctionnement du CTA (conviviaux, délétères, encourageant), les informations sur le soutien familial et/ou associatif (présent, absent, stigmatisation, membre d'une association), les caractéristiques clinico-biologiques (taux de CD4, état général, antécédents, effets secondaires, effets indésirables, fréquence de prise des comprimés, nombre de comprimés pris, traitement associé, consommation des excitants), perception du bénéfice du traitement (positif, négatif, indéterminé), connaissance des conséquences de la NO (oui, non), les principales raisons de la NO. L'exploitation des dossiers médicaux a permis de confirmer les données socio-démographiques, les circonstances de diagnostic, le taux de CD4à l'initiation du traitement et le plus récent, le protocole de traitement en cour ainsi que la date de début de traitement. Les registres de pharmacie ont permis de confirmer le protocole de traitement, la date de début de traitement et évaluer le renouvellement des ordonnances. Une infirmière a été formée dans le cadre de cette étude pour l'appui à la collecte des données.

**Traitement et analyse des données:** les données ont été enregistrées et traitées à l'aide des logiciels Epi Info 7 et Excel 2007. Les données ont ensuite été analysées à l'aide du logiciel XLStat 7.5.2. En analyse bivariée, la comparaison entre les variables qualitatives a été effectuée à l'aide du test de Chi2 de Pearson et la probabilité exacte de Fisher a été déterminée dans le cas de variables dichotomiques. Les différences ont été considérées significatives pour p< 0.05. En analyse multivariée, le modèle de régression logistique a été utilisé pour établir la relation entre la non observance thérapeutique et les variables explicatives. Les variables utilisées pour le modèle de régression logistique étaient celles présentant un p < 0.2. L'Odd ratio et son intervalle de confiance à 95% a été déterminé pour quantifier l'association entre la non observance thérapeutique et les différentes variables explicatives du modèle.

**Considérations éthiques:** l’étude a été soumise et a reçu l'accord du Comité National d'Ethique. L'accord du Directeur de l'hôpital Laquintinie de Douala a également été obtenu pour le recrutement des patients dans cette formation sanitaire. Dans le cadre du recrutement, le consentement des patients a été obtenu après leur avoir expliqué la nature du travail de recherche. Les informations obtenues étaient confidentielles, consignées dans une fiche anonyme.

## Résultats

**Caractéristiques socio-démographiques des patients:** dans cette étude, 603 patients ont été sollicités. Parmi eux, 524 ont répondu (taux de réponse de 86,9%). L’échantillon est composé de 64,6% (n=338) de femmes et 35,4% (n=185) d'hommes (sexe ratio: 0,54). La moyenne d’âge est de 43,0 ans ± 10,7 avec un minimum de 21 ans et maximum de 72 ans. La tranche d’âge de 30 à 44 ans était la plus représentée, soit 49,2% (n=258). Sur les 524 patients inclus dans l’étude, 49,0% (n=257) sont déclarés non observant. Sur les 257 patients NO, 61,9% (n=159) sont des femmes et 38,1% (n=98) sont des hommes (sexe ratio H-F: 0,65), 91,8% (n=236) des patients NO sont de religion monothéiste. En outre, 23,0% (n=59) des patients NO sont sans emploi, 19,1% (n=49) sont commerçants et 16,7% (n=43) des ménagères. La moyenne d’âge des patients NO est de 41,5 ans ± 9,89 et la tranche d’âge de 30 à 44 ans est la plus représentée (51,4%). Pour le statut matrimonial, 38,1% (n=98) de ces patients étaient célibataires, 32,7% (n=84) sont mariés et 9,7% (n=25) veufs. On note également que les niveaux d’études secondaires et supérieures sont les plus représentés, respectivement 56,0% (n=144) et 20,2% (n=52).

**Figure 1:**
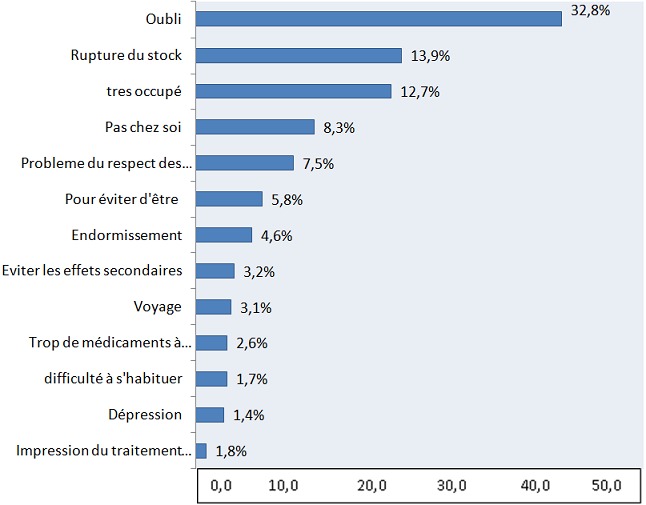
Répartition selon les raisons de la non observance, évoquées par les patients

**Raisons évoquées et facteurs influençant la non observance:** le motif de NO le plus avancé était l'oubli (32,9%). D'autres motifs ont été avancés, notamment la rupture de stock (14,0%), l'occupation (12,8%), le respect des heures de prise (7, 6%) (Figure 1). Concernant les facteurs influençant la NO, les résultats montrent que 57,6% (n=148) des patients NO affirment avoir de la gêne à consommer leurs médicaments (p=0,02). Les patients assujettis à des doses variables d'ARV (plus d'un comprimé par jour), sont majoritaires chez les NO (63,8%) (p= 0,04). La proportion des patients souffrant d'une infection opportuniste est élevée chez les NO (9,0%) (pTableau 1).

**Tableau 1:** Répartition des patients NO selon les facteurs influençant la non observance (n=257)

Variables	Non observant		Observant		
	n	%	n	%	p
Gêne à prendre les médicaments	109	42,4	87	32,5	0,02
Présentation des médicaments					
Dose fixe	93	36,1	120	44,9	
Dose variable	164	63,8	147	55,1	0,04
Infections opportunistes	23	8,9	6	2,2	0,002
NO: non observant					

**Non observance et autres traitements:** les résultats révèlent que 16,6% des patients NO prennent un traitement autre que les ARV. Les guérisseurs représentent plus de la moitié des prescripteurs (51,1%), suivi des médecins 16,3% et des naturopathes 12,8%. En outre, les patients ayant connu des infections opportunistes ont plus tendance à être NO (p=0,02). Pour les autres facteurs, notamment ceux liés à la modification des ordonnances et aux médicaments autres que les ARV, les différences ne sont pas significatives (Tableau 2).

**Tableau 2:** Relation entre non observance et traitement (n=257)

	Non observant n (%)	Observant n (%)	P
Infections opportunistes	23(8,95)	6 (2,25)	0,002
Modification d'ordonnance	126(49,03)	125 (46,82)	NS
Traitement autre que les ARV	48 (18,68)	39 (14,61)	NS

**Relation entre non observance et soutient psycho-social:** en s'intéressant au rapport entre le soutien psychosocial et la NO, on observe que 8,6% des patients NO ont déclaré ne pas avoir reçu un soutien familial contre 3,7% des patients observant (p=0,04). En outre, 72,7% des patients NO ont déclaré percevoir une nette amélioration de leur état de santé, contre 85,4% des patients observant (p=0,006) (Tableau 3).

**Tableau 3:** Relation entre non observance et soutient psycho-social (n=257)

		Nonobservant n(%)	Observant n (%)	P
Bénéficie d'un soutien de la famille		191 (91,3)	236 (96,3)	0,04
Bénéficie d'un soutien social		7 (2,7)	7 (2,6)	NS
Perception de l'amélioration	Aggravation	2 (0,7)	1 (0,3)	
	Aucune amélioration	2 (0,7)	3 (1,1)	
	Faible amélioration	45 (17,5)	21 (7,8)	
	Ne sais pas	21 (8,1)	14 (5,2)	
	Nette amélioration	187 (72,7)	228 (85,3)	0,006

**Association des facteurs de NO en analyse multivariée:** le modèle de régression logistique multivariée associé à ces différents facteurs présente une forte corrélation entre le statut matrimonial (veuf) (IC 95% OR= 1,31-5,22, p= 0,006), la consommation des excitants (IC 95%, OR= 2,30-6,90, p= 0,0001) et la présence d'infection opportuniste (IC 95%, OR= 1,41-17,54, p= 0,01) et la non observance au traitement ARV des patients de cette série (Tableau 4).

**Tableau 4:** Modèle de régression logistique des facteurs indépendamment associés à la non observance.

	modalités	OR	IC à 95% de OR	P
**Profession**	débrouillard	1,0		
	Commerçant	0,881	0,41 - 1,915	0,75
	Employé du privé	0,461	0,207 -1,026	0,057
	Fonctionnaire	0,386	0,13 -1,12	0,079
	Ménagère	0,722	0,33 - 1,60	0,42
	Paysan	8,84	0,84 - 100	0,07
	Retraité	1,971	0,64 - 6,09	0,24
	Sans emploi	1,69	0,77 - 3,70	0,19
**Statut matrimonial**	célibataire	1,0		
	Concubinage	1,384	0,69 - 2,79	0,36
	Divorcé	1,168	0,44 - 3,08	0,75
	Marié	1,07	0,55 - 1,58	0,80
	Veuf	2,62	1,31 - 5,22	0,0062
**Présentation du médicament**	dose fixe	1,0		
	Dose variable	1,45	0,45 -1,05	0,09
Gêne à prendre les médicaments	non	1,0		
	oui	1,39	0,47 -1,11	0,14
soutien de la famille	non	1,0		
	oui	2,027	0,82 - 5,02	0,13
Perception de l'amélioration	aggravation	1,0		
	Aucune amélioration	6,054	0,08 -484,60	0,42
	Faible amélioration	1,16	0,05 - 29,41	0,93
	Ne sais pas	1,477	0,05 - 40,50	0,82
	Nette amélioration	1,777	0,08 -42,00	0,72
**consommation des excitants**	Non	1,0		
	Occasionnellement	1,52	0,41- 1,07	0,09
	Oui	4,03	2,30- 6,90	<0,0001
**infection opportuniste**	non	1,0		
	oui	4,98	1,41- 17,54	0,012

## Discussion

Cette étude s'est fixée pour objectifs de déterminer la prévalence de la NO des PVVIH sous antirétroviraux, de déterminer les caractéristiques socio-démographiques et les facteurs de la NO. Ces objectifs sont proches de ceux de Chkhartishvil et al. qui s’étaient penchés sur l’évaluation de l'adhérence aux TAR dans les pays de l'Europe de l'Est [20].

**Caractéristiques de la non observance:** la NO est associée à plusieurs facteurs, les uns basés sur les déclarations du patient, d'autres liés à la vérification de la régularité des renouvellements des prescriptions en pharmacie, le comptage des comprimés et les variations du taux de CD4. Cependant le comptage des comprimés et les variations du taux de CD4 n'ont pas été pris en compte à cause des ruptures de stocks qu'avait à cette période connu l'hôpital de jour. Ce qui n’était pas le cas pour l’étude similaire menée au Sénégal par Lanièce et al. qui avaient combiné les déclarations du patient et le décompte des comprimés pour l´évaluation de l´observance [15]. Une enquête visant à dresser un état des lieux des recherches et interventions de terrain liées à l´observance dans le domaine du VIH/SIDA en France en 200l, a montré que la majorité des enquêteurs utilisaient exclusivement des indicateurs en rapport avec les dates de renouvellement des ordonnances, la présence aux rendez-vous, du suivi des patients, des marqueurs biologiques (charge virale, taux de CD4) et la vérification des piluliers [21]. De nombreux auteurs sont unanimes qu´il n´existe pas d'instrument de mesure idéal permettant d´évaluer l´observance réelle des patients. Pour pallier à ce déficit, ils préconisent de croiser au moins une méthode objective à une méthode subjective, afin d´obtenir une approximation plus juste de l´observance des patients [6, 22]. Notre étude a le mérite d´avoir combiné une méthode d´évaluation de l´observance subjective à plusieurs méthodes objectives. Un des indicateurs de l’évaluation de la NO est basé sur la période sur laquelle porte la mémoire des patients. Dans cette étude, elle était de 7 jours. La NO des 4 dernières semaines a également été mesurée mais elle n´a pas été utilisée dans les analyses. Dans les cohortes APROCO et MANIFF 2000, les efforts de mémoire portaient sur 4 jours [23]. Dans la cohorte de patients suivis à Dakar, cette évaluation portait sur les 30 derniers jours. Mais les auteurs eux-mêmes sont conscients que cette approche peut apporter des pertes d´informations [15]. Pour plusieurs auteurs, il est souhaitable que les questions sur l´observance portent sur un passé récent (7 jours au maximum) pour minimiser le biais de mémorisation [15, 24, 25]. Dans la présente étude, un patient est classé non observant s´il lui est déjà arrivé d´interrompre volontairement son traitement ou s´il lui est arrivé de sauter une ou plusieurs prises durant les 7 jours précédents la date de l´enquête, ou encore s´il n´a pas respecté un rendez-vous pour le renouvellement de son traitement à la pharmacie. Les déclarations des patients s’étaient avérées propices à l’évaluation de l'observance dans une cohorte sénégalaise [15]. Nous rapportons aussi une proportion de non observance de 49%. Les taux d´observance des traitements antirétroviraux varient considérablement d´une étude à l´autre. Les résultats sont en effet fonction de la méthodologie utilisée pour la mesure de l´observance. Les présents résultats étaient proches de ceux de Mbopi-kéouet al. qui ont trouvé une prévalence de 48,7% de patients NO [16]. Folefacket al. rapportent une proportion de NO de 53,5% [26]. La moyenne d’âge des patients NO de cette étude était de 41,5 ± 9,9 ans, et la tranche d’âge de 30 à 44 ans était la plus représentée. Ce résultat est en phase avec les données statistiques en cours au Cameroun, qui présentent un âge moyen de PVVIH de 43,04 ± 9,88 ans avec la tranche d’âge la plus représentée celle de 30 à 44 ans [27].

**Raisons de la non observance:** les dix principales raisons de non observance évoquées sont: l'oubli; la rupture de stock; l'occupation; ne pas être chez soi; le respect des heures de prise; le fait d’être vu consommant des médicaments; le sommeil; les effets secondaires; l'importance du nombre de médicaments. Mbopi-kéou et al. ont trouvé par ordre d´importance: l´oubli, le travail, l´endormissement, la mobilité (voyage), l´absence de nourriture. La rupture de stock à la pharmacie en était la 7ème cause et la prescription inadéquate occupait le 9ème rang [16]. Dans une enquête longitudinale en milieu hospitalier à Bamako, Oumar et al. rapportent que l´oubli constitue le principal facteur de mauvaise observance [28]. Afionget al. en 2011 ont trouvé pour principales causes l'occupation; l'oubli et la dépression [29]. Ces résultats sont différents de ceux observés dans les pays occidentaux ou les motifs liés aux médicaments sont la première cause de non-observance [30]. L´engagement du patient lui-même à suivre son traitement semble être le principal déterminant de l´observance, suivi des facteurs institutionnels comme l´effet des ruptures de stocks en médicaments et des prescriptions inappropriées.

**Caractéristiques des patients non observant:** la NO est fortement associée à l’âge et au statut matrimonial. Les patients de plus de 60 ans présentent une meilleure observance que ceux de 30 à 44 ans. Les personnes veuves sont plus observantes que les célibataires. Des travaux ont effectivement démontré une variabilité importante des caractéristiques socio-démographiques associés à la NO [31]. Cette variabilité peut s´expliquer d´une part par les différences au niveau des populations étudiées, et d´autre part par la méthode utilisée. Spire B. et al. dans l´étude de la cohorte APROCO, trouvent uniquement l´âge comme seule variable sociodémographique associée à l´observance. Les patients les plus âgés ont tendance à rester plus observant que les plus jeunes. Ils ne trouvent aucune association avec le genre, l´emploi ni le niveau d´étude [23]. Les patients ressentant une gêne à prendre les médicaments, ceux qui prennent des doses variables et ceux qui ont eu des infections opportunistes, sont significativement plus non observant. AfiongOkuet al. au Nigeria ont démontré que le nombre de comprimés (plus de 2 par jour) est un facteur significatif de la NO [29]. On note que ceux qui prennent un traitement autre que les ARV ne sont pas plus non observant. Ce qui n’était pas le cas pour Afionget al. au Nigeria qui ont montré que la non utilisation des plantes médicinales est fortement associée à une bonne observance [29]. Mbopi-kéouet al. ont eux démontré que les patients ayant eu recours au traitement traditionnel après le début du TAR apparaissent moins observant que ceux sous TAR exclusif [16]. Une association entre le soutien familial et la NO est notée dans cette étude. La cohorte APROCO révèle que le manque de soutien social et familial est significativement associé à une mauvaise observance [23]. Par ailleurs, les patients qui ont perçu une nette amélioration de leur état de santé durant le processus de traitement sont plus observant que ceux ayant perçu une faible amélioration.

**Limites de l’étude:** au regard des objectifs de cette étude, les difficultés observées ont été liées au problème de l’étude de l'observance proprement dite. En réalité, il n'existe pas de méthodes référence en matière d'observance [32] et il est recommandé de recourir à au moins deux méthodes dont l'une repose sur la déclaration du patient [25]. Le patient a cependant toujours tendance à surestimer son observance. Cette étude a également été confrontée à l'absence de prise en compte de méthodes biologiques telles que la mesure de la charge virale et le dosage des anti-protéases qui auraient amélioré la validité des résultats [33]. Certains auteurs suggèrent la nécessité de valider la méthode utilisée pour estimer l´observance, en mettant en évidence une corrélation entre le niveau d´observance et la réponse virologique des patients. Malheureusement, l´évaluation de la charge virale n'a pas été faite à cause du coût de cet examen dans le contexte du Cameroun. Il aurait certainement été possible de le corréler avec le taux de CD4 qui était indisponible au moment de l’étude. Afin de limiter les biais de mémorisation, nous avons évalué auprès de ces patients, l´observance au cours des sept derniers jours et du mois précédents le début de l´enquête. Ce qui n´exclut cependant pas la possibilité de biais de mémorisation (perte d'information de la part du patient concernant le respect de sa prescription). Par ailleurs, certaines études ont révélé que les patients en présence de l´enquêteur sont parfois emmenés à faire de fausses déclarations [34, 35].

## Conclusion

Les facteurs associés à la non observance ont été identifiés à savoir: le statut matrimonial (veuf); la consommation des excitants et les infections opportunistes. Au vue de la diversité de ces facteurs, il serait judicieux d'insister sur le renforcement des éducations thérapeutiques, la prise en charge psychosociale des patients sous traitement ARV et la limitation des ruptures de stocks. Seule la gratuité des antirétroviraux ne suffit pas à résoudre les problématiques de la non observance.
